# High-resolution 3D photopolymerization assisted by upconversion nanoparticles for rapid prototyping applications

**DOI:** 10.1038/s41598-018-21793-0

**Published:** 2018-02-26

**Authors:** Vasilina V. Rocheva, Anastasia V. Koroleva, Alexander G. Savelyev, Kirill V. Khaydukov, Alla N. Generalova, Andrey V. Nechaev, Anna E. Guller, Vladimir A. Semchishen, Boris N. Chichkov, Evgeny V. Khaydukov

**Affiliations:** 1 Federal Scientific Research Centre “Crystallography and Photonics” of Russian Academy of Sciences, Moscow, 119333 Russia; 2 Laser Zentrum Hannover, Hannover, 30419 Germany; 3 Shemyakin-Ovchinnikov Institute of Bioorganic Chemistry of the Russian Academy of Sciences, Moscow, 117997 Russia; 4 Institute of Fine Chemical Technologies, Moscow Technological University, Moscow, 119571 Russia; 5 Institut für Quantenoptik, Leibniz Universität Hannover, Hannover, 30167 Germany; 6 Sechenov First Moscow State Medical University, Moscow, 119991 Russia; 7 Macquarie University, Sydney, NSW 2109 Australia; 8 International Joint Center for Biomedical Innovation, School of Life Sciences, Henan University, Kaifeng, Henan 475004 China

## Abstract

Three-dimensional (3D) rapid prototyping technology based on near-infrared light-induced polymerization of photocurable compositions containing upconversion nanomaterials has been explored. For this aim, the rationally-designed core/shell upconversion nanoparticles NaYF_4_:Yb^3+^,Tm^3+^/NaYF_4_, with the distinct ultraviolet-emitting lines and unprecedentedly high near-infrared to ultraviolet conversion efficiency of ${\eta }_{{\bf{UC}}}^{({\bf{UV}})}=2{\boldsymbol{ \% }}$ have been used. The upconverted ultraviolet photons were capable to efficiently activate photoinitiators contained in light-sensitive resins under moderate intensities of NIR excitation below 10 W cm^−2^ and induce generation of radicals and photopolymerization *in situ*. Near infrared-activated polymerization process, both at the millimeter and sub-micron scales, was investigated. Polymeric macro- and microstructures were fabricated by means of near infrared laser scanning photolithography in the volume of liquid photocurable compositions with focused laser light at 975 nm wavelength. Examination of the polymerization process in the vicinity of the nanoparticles shows strong differences in the rate of polymer shell growth on flat and edge nanoparticle sides. This phenomenon mainly defines the resolution of the demonstrated near infrared - ultraviolet 3D printing technology at the micrometer scale level.

## Introduction

Photopolymerization processes such as direct laser writing, three-dimensional (3D) micromachining, holography, micro- and optoelectronics, optical element formations, data recording and storage, etc. are widely used in various fields of science, industry and technology^1^. Typically, it implies transformation of a liquid mix of a photopolymerizable monomer or cross-linkable polymer and a photoinitiator (termed as a photocurable composition, or PCC) into a solid material under light irradiation. Light-based triggering is the key advantage of this technology that allows high spatial and temporal resolution achieved in a non-contact manner. The single-photon polymerization is usually activated by irradiation with ultraviolet (UV) or short-wavelength visible light, but without the repetition steps of adding of the subsequent polymer layers, it is mainly limited to the fabrication of two-dimensional (2D) structures. This shortage stems from the following time and spatial restrictions. First, the strong light absorption by photoinitiator results in stable radical formation and immediate triggering of the polymerization process^2^. In addition, linear light absorption does not allow direct production of bulk 3D structures of consistent density because the UV and visible light penetration depth is limited to a near-surface layer due to the exponential light attenuation^3^.

Several strategies have been developed to increase the depth of photopolymerization and 3D structure formation. In the mid-1980s, C. Hull invented the stereolithography (SLA) technology, relying on layer-by-layer fabrication of well-defined 3D patterned structures by exposing the UV-curable materials to a scanning laser beam^4^. 3D two-photon photopolymerization process represents the next generation of the technology. This technology benefits from the fundamental property of nonlinear light absorption by a photoinitiator under high-intensity laser radiation^5,6^. Shifting to a near-infrared (NIR) light as a photo-trigger has led to the increased light penetration depth^7^ and allowed drawing of 3D structures directly in the volume of a PCC^8^. The ultimate resolution of this method is determined by the properties of the PCC with the voxel size limited at approximately 100 nm^9^. However, the expensive instruments (e.g., femtosecond lasers), high laser intensities, and time-consuming spot-by-spot curing process hinder broad applications of this technology^10^.

The application of lanthanide-doped upconversion materials (UCMs) allows overcoming of many problems associated with using of NIR light as the irradiation source for the photopolymerization process^11^. The UCMs sequentially absorb several NIR photons through the long lifetime and ladder-like energy levels of trivalent lanthanide ions (ytterbium, erbium or thulium), co-embedded in an inorganic host matrix^12^. This results in anti-Stokes, or upconversion, luminescence^13^. The spectral band of the excitation light, centered at 975 nm, corresponds to the low-loss optical window of commonly used polymer materials, where light penetrates in PCC with minimal absorption and scattering. The nonlinear character of the upconversion process provides a voxel formation^14,15^ that enables the fabrication of 3D structures directly in the volume of PCC. Furthermore, upconversion luminescence can be excited with a moderate intensity of a few W cm^−2^ by affordable semiconductor lasers^13,16^.

The upconversion nanoparticles (UCNPs) composed by NaYF_4_ ceramic host co-doped with Yb^3+^ as a sensitizer, and Er^3+^ or Tm^3+^ as activator, are considered as one of the most efficient anti-Stokes photoluminescent materials^17^. These nanoparticles demonstrate upconversion emission with narrow lines in UV and visible spectral ranges under continuous-wave (CW) excitation at 970–980 nm. In addition, these nanoparticles combine a large anti-Stokes shift of several hundred nanometers, non-photoblinking nature, and superior photostability.

To date, only a few reports demonstrate NIR-light-activated photopolymerization mediated by upconversion emission. The idea of planar upconversion lithography has been demonstrated^18^. UCNP based photoinitiator systems and radical polymerization driven by NIR-light were shown^7,19^. NIR light converted to the green luminescence of NaYF_4_:Yb^3+^, Er^3+^ nanoparticles, has been successfully used to induce straightforward thin polymer shell formation on UCNP surface for its hydrophilization^20^ and drug loading^21^. Micrometer-sized UCMs were used for NIR-induced curing of PCC^22–24^. In particular, recently J. Méndez-Ramos *et al*. provided qualitative analysis of feasibility of 3D structures formation induced by NIR-to-UV upconversion photoluminescence with the use of very large Tm^3+^-doped K_2_YbF_5_ crystals^23^. Liu *et al*. demonstrated deep photopolymerization (more than 10 cm) of PCC containing upconversion nanoparticles in the glass tube, which was vertically exposed to a fiber coupled 980 nm laser^10^. However, these studies left unexplored the key aspects of UCM-assisted 3D structure formation, including high spatial resolution and controllability of the 3D-photocuring processes under reduced power of the excitation light, which are crucial for further development and implementation of this innovative technology.

In this paper, we investigate the feasibility of NIR-induced polymerization of PCC containing UV-emitting upconversion nanomaterials under conditions of direct writing by semiconductor laser for the 3D rapid prototyping applications. We report on the rational design of UCNPs to yield unprecedentedly high conversion efficiency in the UV range. Unique luminescent properties of the synthesized nanomaterials enable us to demonstrate the NIR-induced polymerization process both at macro- and micrometer scales.

## Results

**Figure 1:**
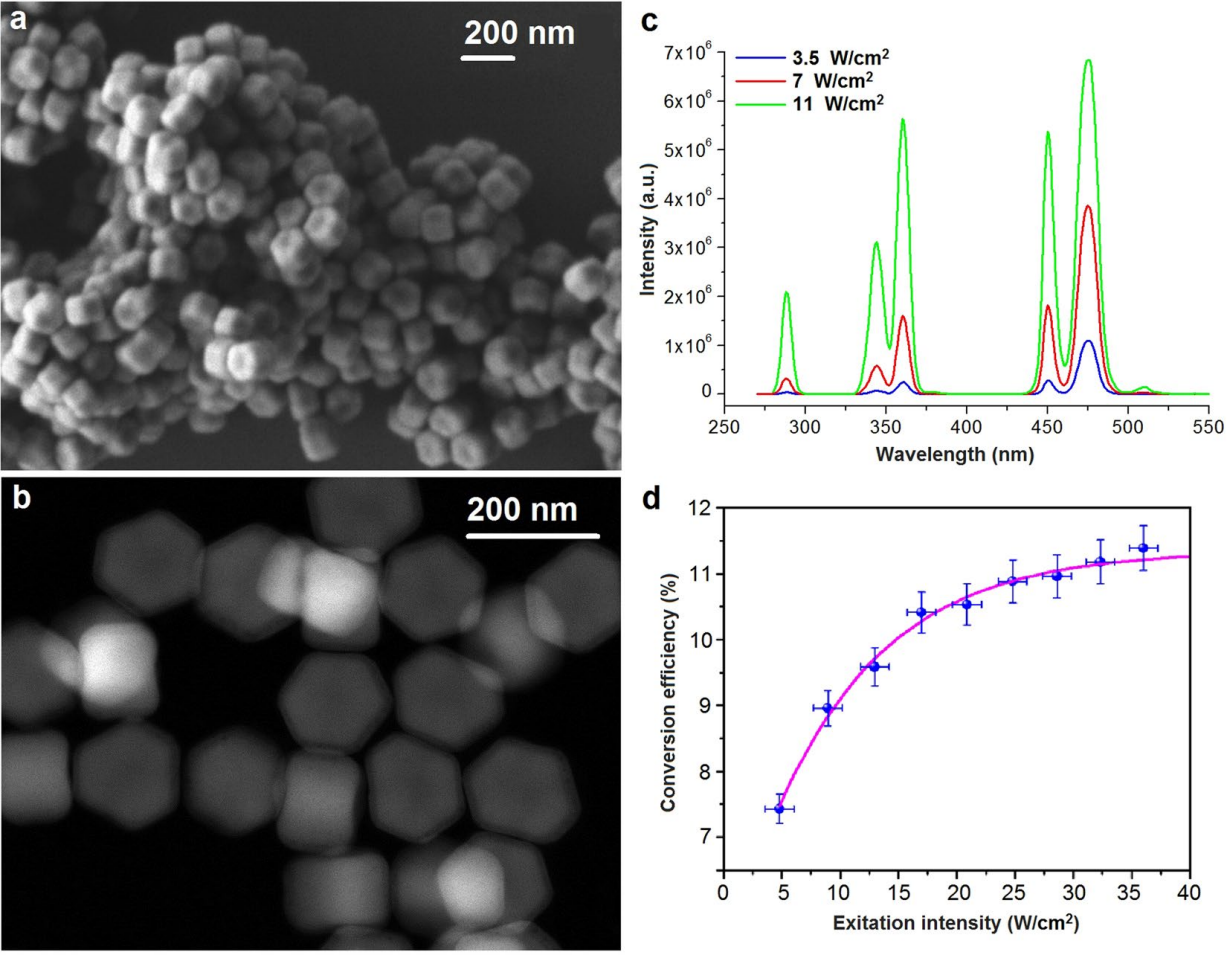
Characterization of the synthesized UCNPs. (**a**) SEM and (**b**) high-angle annular dark-field (HAADF) scanning TEM images of *β*-NaYF_4_: Yb^3+^,Tm^3+^/NaYF_4_ core/shell nanoparticles. (**c**) Spectra of the UCNPs in chloroform irradiated with 975-nm laser light with the intensity of 3.5, 7 and 11 W cm^−2^. (**d**) Integral conversion efficiency of UCNPs versus the excitation intensity at 975 nm measured using the calibrated integrating sphere setup. The saturation was reached at ∼20 W cm^−2^. The magenta line is provided as a guide to the eye.

The core/shell *β*-NaYF_4_:Yb^3+^,Tm^3+^/NaYF_4_ UCNPs were synthesized in order to serve as an internal NIR-triggered UV source for photopolymerization at a considerable depth. The core size of the particles was 150 nm × 100 nm (Fig. 1a,b), while the thickness of the inactive NaYF_4_ shell was 5 nm allowing the surface defect passivation and elimination of the influence of the quenching factors of the liquid microenvironment^25^. The rather large diameter of the core (≥100 nm) was specially chosen to obtain distinct spectral bands of the UCNPs in UV range at the relatively low (<100 W cm^−2^) excitation intensities of NIR light^26^. Figure 1c demonstrates photoluminescence (PL) spectra of UCNPs excited by a CW laser at the wavelength of 975 nm. The PL spectrum of the prepared UCNPs featured five emission bands, which are known to result from the Tm^3+^ electronic transitions^27^. In particular, the following narrow luminescent lines in UV bands, corresponding to Tm^3+^ transitions ^1^I_6_ →^3^H_6_ (288 nm), ^1^I_6_→^3^F_4_ (345 nm) and ^1^D_2_→^3^H_6_ (360 nm), were observable.

The intensity of ion transitions in the anti-Stokes luminescence spectrum is characterized by a power-law dependence of the exciting intensity^28^. For example, thulium ion transition ^1^D_2_→^3^H_6_ (360 nm) can be described by number *n* = 4 of the excitation quanta (975 nm), which are required for the thulium ion excitation at the state ^1^D_2_. It means that the emission intensity (*I*_em_) at a wavelength of 360 nm, corresponding to this transition, is approximately proportional to the fourth power of excitation intensity (*I*_ex_). Tm^3+^ transitions ^1^I_6_ →^3^F_4_ (345 nm) and ^1^I_6_→^3^H_6_ (288 nm) can be realized at *n* = 5 and *n* = 6 of the excitation quanta (975 nm), respectively. A value of ${\eta }_{{\rm{UC}}}^{({\rm{UV}})}$ = 2.0% at the excitation intensity of 25 W cm^−2^ was achieved, while the integral *η*_UC_ reached 10.5 ± 0.5% at the same excitation intensity (Fig. 1d). The full emission spectrum of UCNPs is detailed in the Section S1, Supplementary Information.

**Figure 2:**
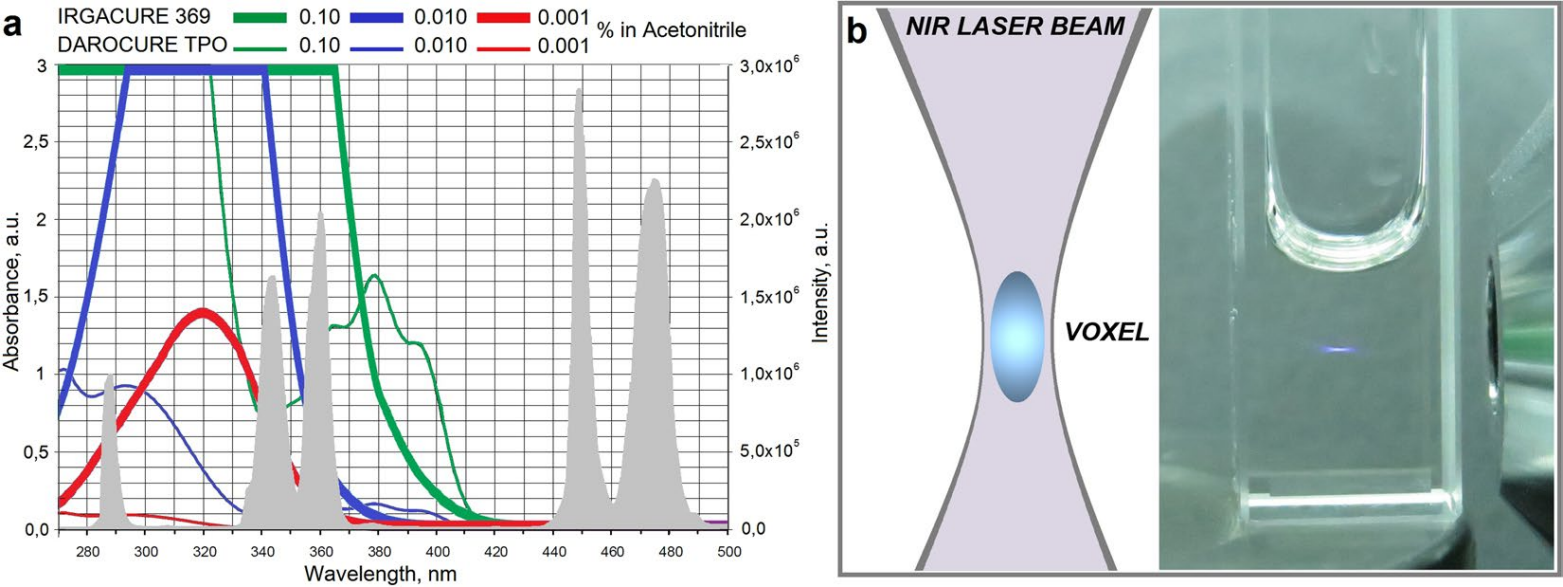
(**a**) Overlap of the absorption spectra of photoinitiators Irgacure 368 and Darocure TPO in acetonitrile^37^ and the emission spectrum of the UCNPs *β*-NaYF_4_:Yb^3+^,Tm^3+^/NaYF_4_ under NIR excitation at 975 nm at intensity 15 W cm^−2^ (shown by grey peaks). (**b**) Luminescent voxel formation in 10 mm × 10 mm cuvette containing light-sensitive resin impregnated with UCNPs under CW NIR light illumination at 15 W cm^−2^ intensity.

To start the cross-linking and photopolymerization processes, the photoinitiator should be activated by radiation at a wavelength that falls within its absorption spectrum. As a result, a stable radical, capable to photoinitiate cross-linking, is generated. The upconversion photoluminescence spectrum of as-synthesized UCNPs (at 288, 345 and 360 nm wavelengths) was anchored within the absorption band of the chosen photoinitiators Irgacure 369 and Darocure TPO (Fig. 2a).

Next, the examination of the luminescent voxel formation and the spatial resolution of the UCNP-mediated NIR-triggered photopolymerization were performed. Following the experimental data, focusing of the laser beam into a medium, containing upconversion nanoparticles, makes it possible to localize of the luminescence region into a voxel, similar to the two-photon excitation method (for details see Section S2, Supplementary Information). A considerable reduction of the minimal voxel size was observed under a relatively low NIR intensity (Fig. 2b). In contrast to two-photon photopolymerization^29^, the substantially lower intensities of the exciting radiation for a voxel formation were required, as the excitation occured through real energy states. However, note, that the effective voxel size in NIR-induced cross-linking will be higher, compared to the two-photon process. Generally, the following scenarios occur due to three excitation regimes^30^: the low power (*I*_em_ ~ [*I*_ex_]^n^), the high power (when the slope declines with the number of photons involved) and the saturation regime (*I*_em_ ~ [*I*_ex_]).

**Figure 3:**
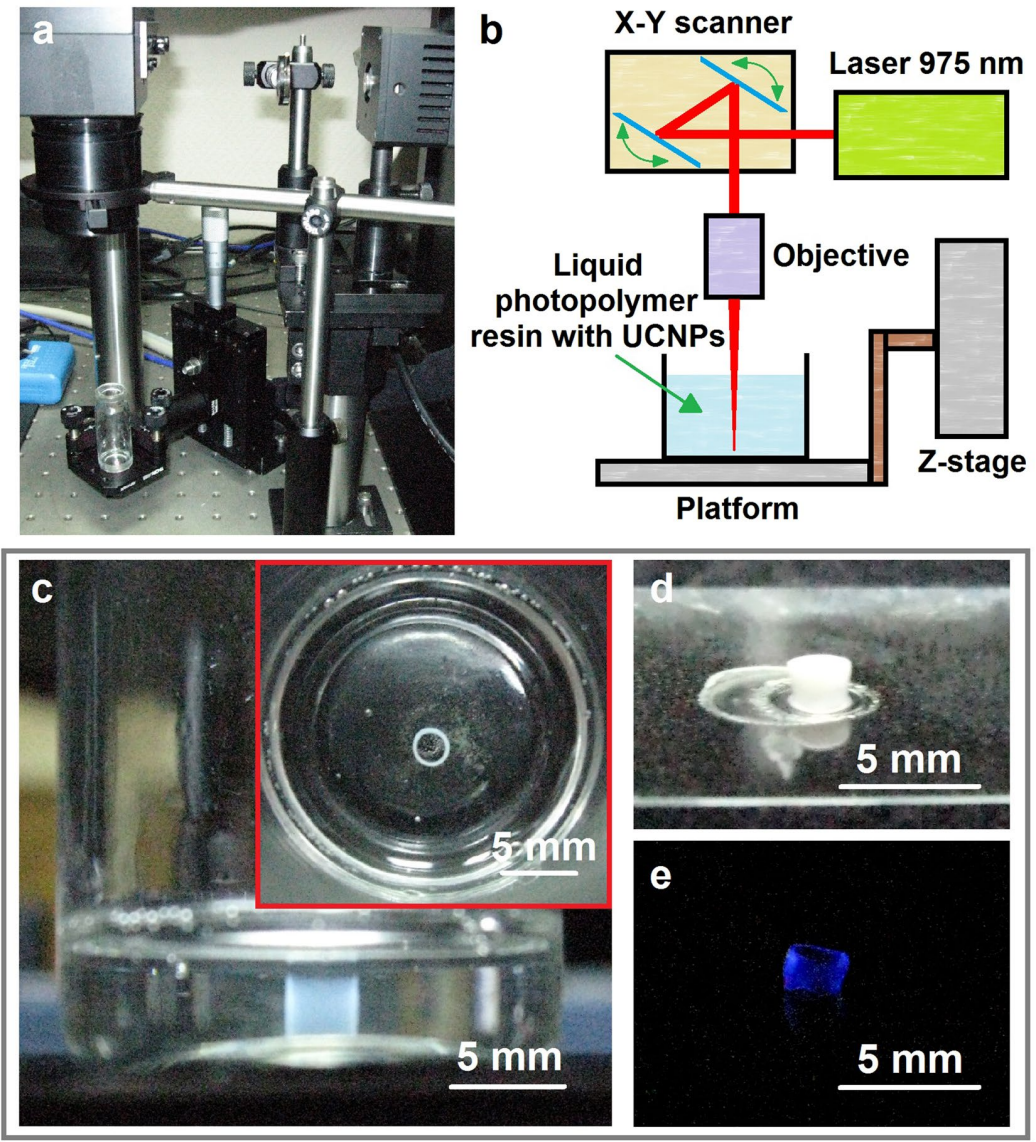
(**a**) The experimental setup for fabrication of 3D polymeric structures in PCC containing UCNPs under NIR irradiation and (**b**) its scheme. (**c**) Top-view image of a structure produced by NIR triggered 3D photopolymerization, (**d**) a 3D structure obtained via NIR-triggered photopolymerization after developing and (**e**) its anti-Stokes luminescence under 975 nm excitation.

We successfully carried out the formation of 3D polymer structures in PCC volume, demonstrating a cost-effective UCNP-assisted 3D printing technology. Commercial CW laser diode at 975 nm (ATC-SD, Russia) was focused by an objective in PCC volume, containing UCNPs. The power density of the laser was set at 15 W cm^−2^ (Fig. 3a,b). As a proof of feasibility of the proposed approach for creation of the 3D printed device macroarchitecture, the hollow tube formation with the diameter of 1.5 mm and the height of 2 mm was obtained by layer-by-layer drawing directly in the resin volume (Fig. 3c,d). The PCC was scanned by the laser via 2-axis galvano-scanner Miniscan-07 (Raylase, Germany). The displacement of voxel along the z-axis was carried out by a micrometric translation stage (Thorlabs). After finishing of the drawing process, the tube was removed and developed in 2-propanol. Note, that the final polymer product exhibited visible luminescence under NIR irradiation (Fig. 3e).

**Figure 4:**
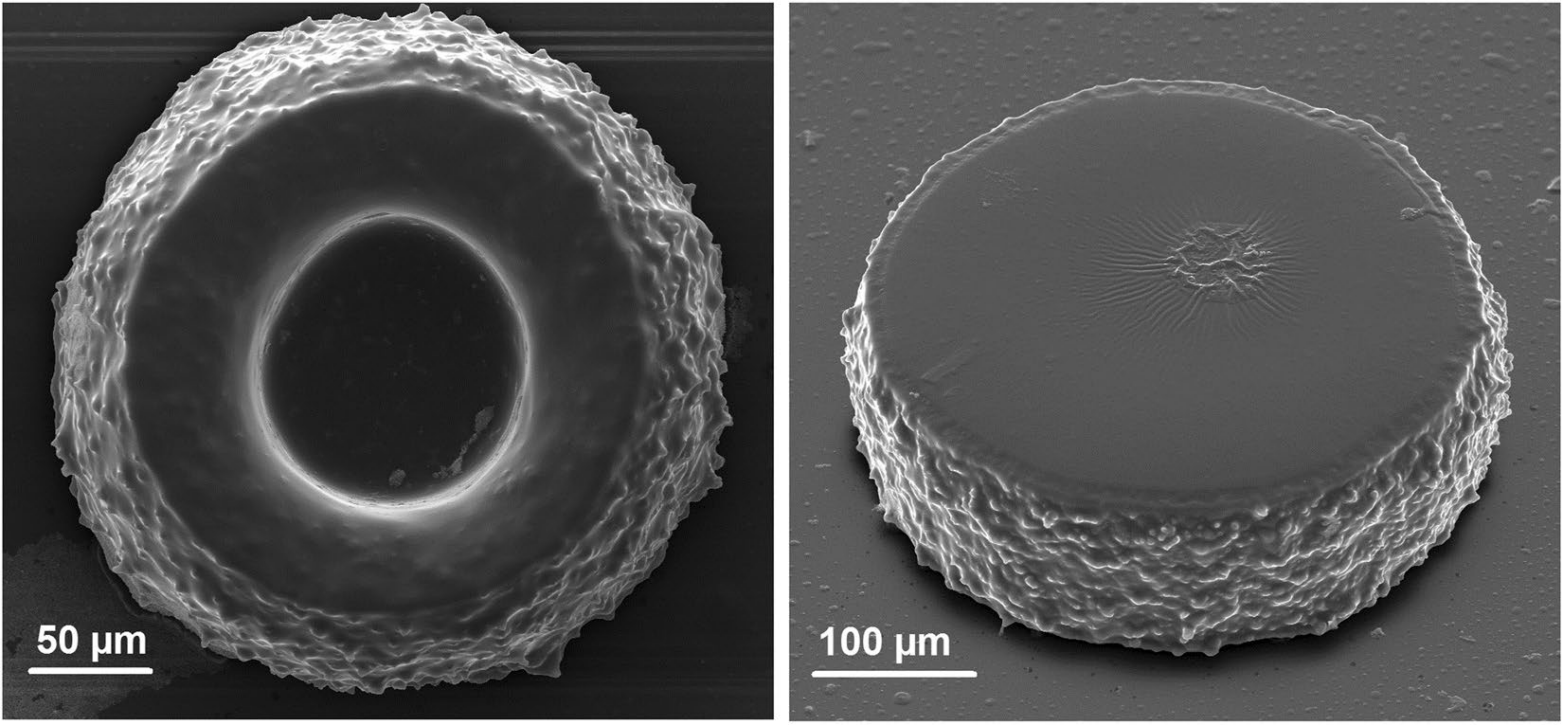
SEM images of 3D polymer microstructures obtained by NIR-light-activated photopolymerization in a thin layer.

In order to demonstrate the microstructure formation, we equipped the experimental setup with a microscope objective lens (20×) and motorized z-axis translation stage. A 50 µL drop of UCNP-containing PCC was placed between the microscope coverslips positioned at the distance of 100 µm from each other by an uncompressible spacer. NIR irradiation was started 30 minutes after the sample preparation when the vortex motion of the liquid material in the layer had stopped. The structure formation started from the glass surface and was performed by means of layer-by-layer drawing in PCC with 10 µm z-axis steps. Continuous laser beam scanning in the X-Y-plane with an estimated speed of 0.2 mm s^−1^ was realized by 2-axis galvano-scanner. The power density of the laser was 7 W cm^−2^. After the drawing process, the assembly of the coverslips was separated and the resulting products were developed in 1-propanol. Figure 4 shows scanning electron microscopy (SEM) images of the fabricated structures.

**Figure 5:**
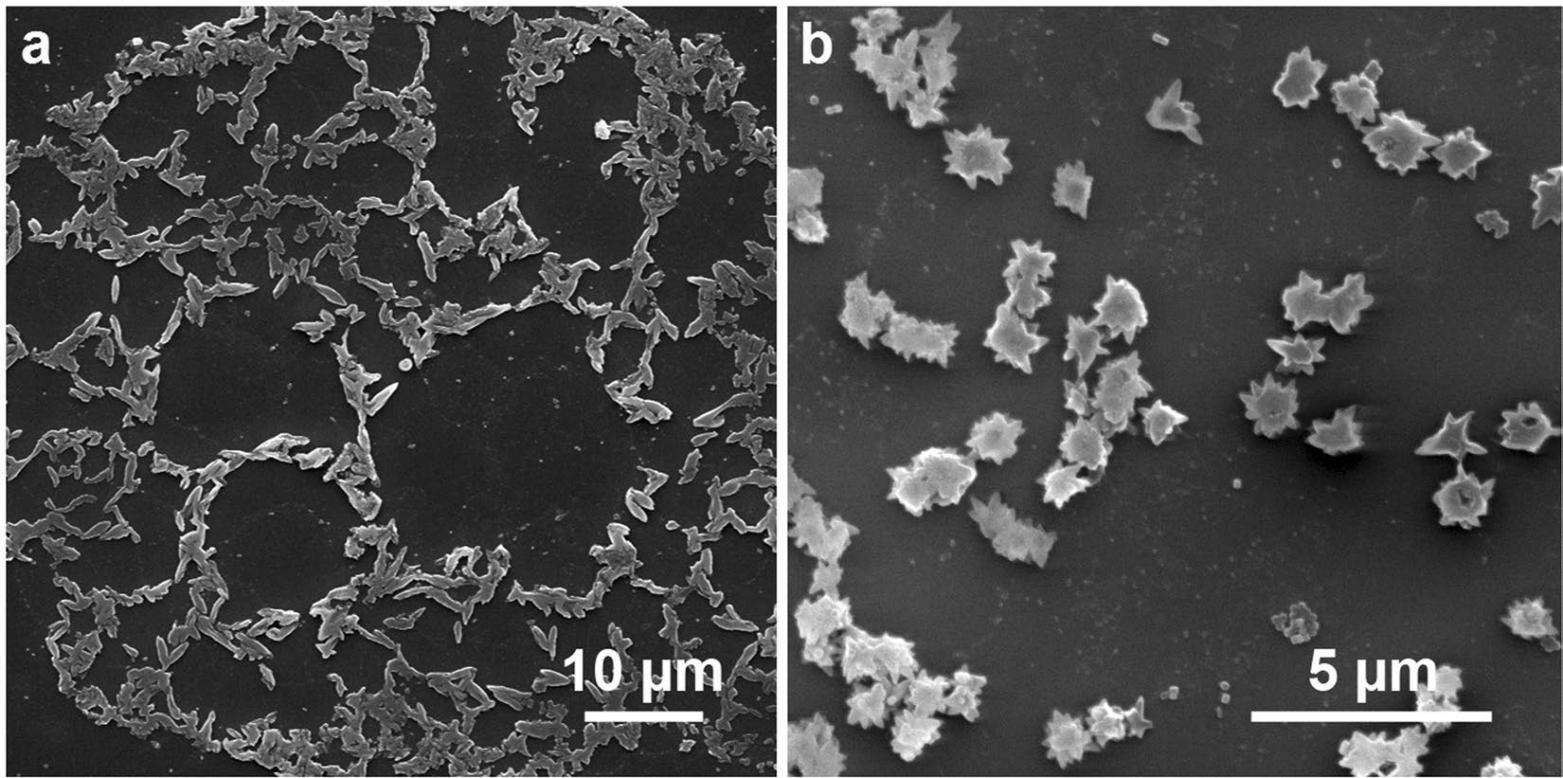
Morphology of photopolymerized PCC around UCNPs under 975 nm irradiation: (**a**) Formation of rice-like structures around single UCNPs; and (**b**) star-shaped clots around UCNP clusters.

Figure 4 demonstrates high roughness of the sides of fabricated microstructures. This can be explained by the peculiar effects observed during the polymer structure growth around the surface of single nanoparticles. To demonstrate this phenomenon, a thin layer of UCNPs was placed on the glass coverslip, covered by a thin film of photosensitive monomer and exposed to IR irradiation. Figure 5 demonstrates the rice-like structures with an average size of 850 nm × 350 nm ± 50 nm obtained by this procedure.

Formation of rice-like structures indicates different polymer growth kinetics on the flat and the narrow edge surfaces of single UCNPs. We believe that this effect is determined by the heterogeneous distribution of photoluminescence intensity around UCNP. Luminescence from crystals is often anisotropic. Chen *et al*. demonstrated the polarization behavior of upconversion luminescence from single NaYF_4_ nanodisk^31^. Green *et al*. found that anisotropic shaped nanoplasmonic UCNPs, oriented with flat configuration, showed stronger emission intensity than those with edge orientation^32^. Calculations show that electric field strength is determined by the geometry of the UCNP. Our experiments on the formation of rice-like structures around the UCNP allow “fixation” of the near-field distribution. The geometric profiles of the obtained rice-like structures indicate that the intensity of the emitted field from the top and bottom faces of UCNP is approximately four times higher than the intensity of the emitted field from the edge sides.

In order to understand the threshold nature of nanoparticle concentration in photocurable composition required for 3D structure formation, we describe it from the point of view of percolation theory. The mathematical model of percolation has a geometrical-statistical character. The percolation process is defined by assigning some random mechanism to the medium. The model of percolation assumes that a permeable matrix is randomly filled with objects in free space overlaps. More objects, added to the matrix, eventually result in a geometrically connected phase^33^.

**Figure 6:**
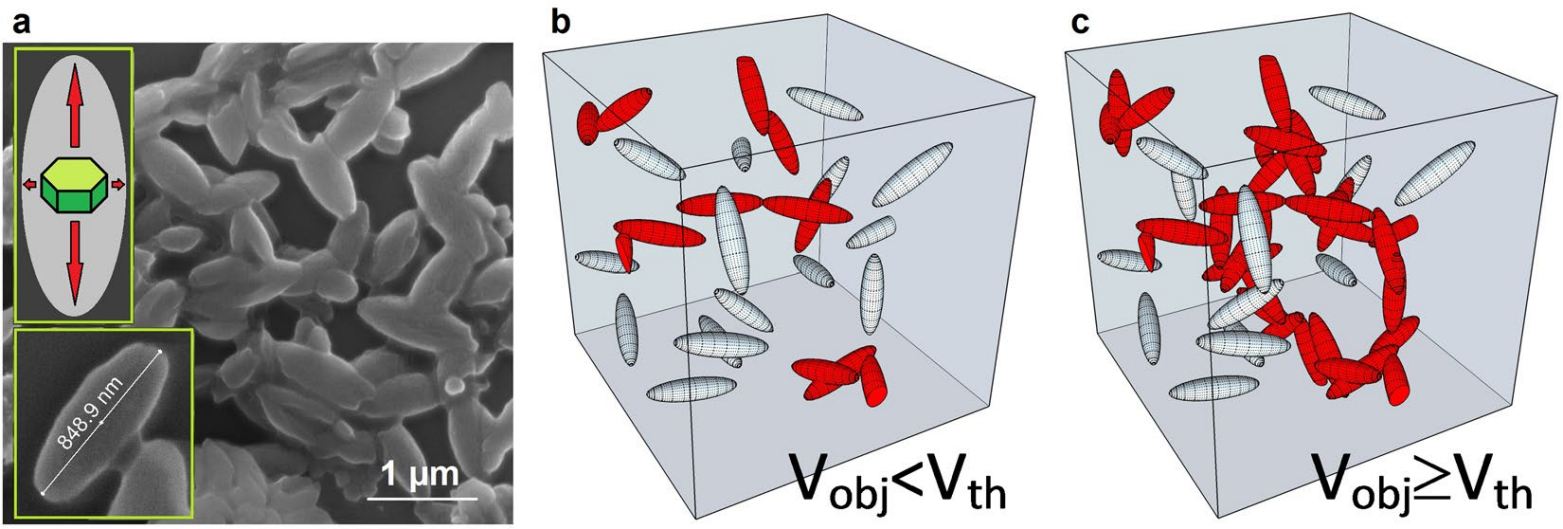
Concentration threshold nature of nanoparticle-induced photopolymerization for 3D structure formation. (**a**) SEM image of rice-like structures. (**b**,**c**) Formation of geometrically connected phase in PCC matrix. Red ellipsoid objects determine the spatially connected phase in the matrix. Grey ellipsoids are objects without binding.

Indeed, the formation of 3D structures is only possible by the formation of geometrically connected polymeric objects growing from nanoparticle surfaces. This fact limits the required minimum concentration of nanoparticles within the photocurable compositions. Lowering UCNP concentration in the volume of photoresin below the threshold requirement leads to the formation of local polymer clusters, making it impossible form of stable, spatially connected 3D structure (see Fig. 6b,c).

For the formation of geometrically connected phase in the matrix, the volume randomly filled by elementary objects should overcome a certain critical threshold *V* ≥ *V*_th_. The threshold value of the filled volume *V*_th_ is determined by the shape of the objects, elementary object interaction, matrix lattice and dimensions of this lattice. In our case, the elementary object can be represented as an ellipsoid with the geometric aspect ratio of 1/3 (see insert in Fig. 6). The critical volume fraction of ellipsoids with 1/3 aspect ratio was calculated by Yi and Sastry^34^, and was found to be ≈20 vol.%.

To determine the critical concentration of (UCNPs) required for 3D structuring, the volume of the formed polymeric rice-like shell (*V*_obj_) and the volume of UCNP (*V*_ucnp_) should be compared:

$$\frac{{V}_{obj}}{{V}_{UCNP}}\approx 50.$$

Therefore, the critical volume fraction of UCNPs needed for the photocomposition is 50 fold lower and corresponds to 20 vol.%/50 ≈ 0.4 vol.%.

According to Liu *et al*.^35^ the UCNP density is *ρ* = 4.2 g cm^−3^. Thus, the threshold of UCNP concentration in photosensitive composition for 3D structure formation during polymerization is ≈16.8 mg ml^−1^.

In fact, the UCNP concentration below the threshold does not mean that the process of photopolymerization is impossible. However, the character of this process will be changed in such manner that the nanoparticles will form polymeric shells around themselves. The thickness of these shells may be determined by the photon mean free path in the photosensitive composition (${l}_{{\rm{PI}}}=1/{\alpha }_{{\rm{PI}}}=1/({\varepsilon }_{{\rm{PI}}}\times {C}_{{\rm{PI}}})$, where *α*_PI_, *ε*_PI_, *C*_PI_ denote the linear absorption coefficient, molar extinction coefficient and molar concentration of photoinitiator, respectively). At a sufficient UV photon flux from UCNPs, and taken that the photon mean free path in the medium exceeds the half of the mean distance between the UCNPs, the polymeric shells, formed around UCNPs will start to fuse, leading to the linked volume formation. Though, the spatial resolution in this case will be determined by the mean free path of UV photons irradiated by UCNPs in the photosensitive medium.

**Figure 7:**
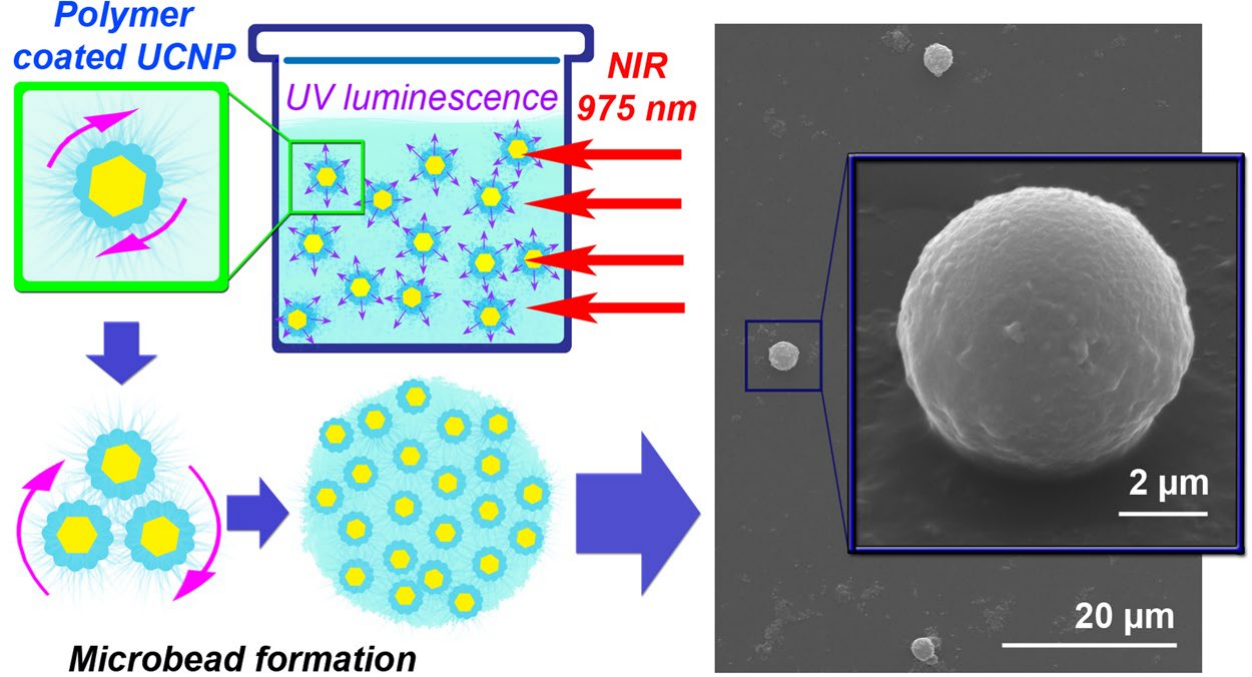
Formation of polymeric microbeads in a photosensitive composition containing UCNPs with the concentration of 0.15 mg ml^−1^ which is below the threshold value.

Figure 7 demonstrates that IR exposure of a photosensitive material containing UCNP inclusions below the threshold value leads to the formation of polymeric microbeads in the PCC volume. We think that these structures are formed due to the nanoparticle aggregation induced by their movement in the gradient of the thermal field generated during the irradiation process. Taking into account the nanoparticle aggregation, its cumulative radiation profile corresponds to that of a sphere with the following spatial intensity distribution *I* = *I*_0_/*R*^2^. Such distribution of the UV intensity provides the production of polymeric spherical microbeads.

It should be emphasized that the proposed methodology can be adapted for a number of applications requiring initial delivery of a liquid materials to the subsurface and confined spaces with following solidification and shaping. The combination of the NIR-light triggering with the UCNP-based UV-emission allows “delivery” of the triggering irradiation to PCC to the certain depths. At the same time, the low intensities of the used NIR laser light opens the opportunity to translate this technique for various biomedical applications. For example, it is considered that such a kind of direct writing maybe useful for the macro- and microstructural configuration of various implantable devices. However, the biocompatibility and biosafety of the materials used for creation of PCC should be specially addressed before the *in vivo* testing.

We also suppose that the unique growth behavior of the PCC in vicinity of the UCNPs during photopolymerization may serve as a promising visualization tool for the studies of the complex aspects of UCNP photophysics.

## Conclusion

In conclusion, we demonstrated the effective and straightforward strategy for the NIR-activated polymerization of photocurable compositions containing UV-emitting upconversion nanomaterials. The core/shell upconversion nanoparticles NaYF_4_:Yb^3+^,Tm^3+^/NaYF_4_, were rationally designed to provide UV-emitting lines at unprecedentedly high NIR-UV conversion efficiency. Their ability to activate commercially available photoinitiators in the process of radical polymerization of light-sensitive resins at moderate NIR light intensities and irradiation doses opens unique possibilities for the implementation of novel NIR triggered photopolymerization technique. This technique, similar to two-photon polymerization, allows fabrication of 3D polymeric structures inside the volume of photocurable compositions. Studies of polymerization process in the vicinity of nanoparticles resulted in the demonstration of the strong difference in the morphology of polymer shell formation on the flat and edge nanoparticles’ sides. This phenomenon, illustrating complexity and richness for the UCNP photophysics, defines the resolution of the demonstrated NIR-UV 3D printing technology at millimeter and sub-micrometer scales. The analysis of the physics foundations of the proposed methodology indicates that it could be efficiently applied for the non-contact control of formation of 3D shapes in depth of the other materials, including the biotissues.

The demonstrated UCNP-assisted photopolymerization process is promising for the development of novel functional polymer composites and high-speed generation of 3D polymeric structures featured with macro- and microarchitecture. This technique is highly versatile and translatable for the applications in industry and biomedicine.

## Methods

### UCNP Synthesis

All chemicals were purchased from Sigma-Aldrich (Germany). The lanthanide-doped NaYF_4_ nanocrystals were synthesized via the coordinate stabilization of yttrium, ytterbium, and thulium metal salts in a solution of oleic acid and octadecene carried out with heating at a rate of 200 °C min^−1^ up to 320 °C in a Wood’s alloy bath in an oxygen-free atmosphere. The details of UCNP synthesis were described by us elsewhere^27^. The core formed by a nanocrystal of *β*-NaYF_4_ co-doped with the Yb^3+^ and Tm^3+^ ions in 18% and 0.6% molar ratios, respectively; was coated with an undoped crystal shell of NaYF_4_ resulting in a core-shell structure of *β*-NaYF_4_:Yb^3+^,Tm^3+^/NaYF_4_. The products of the synthesis were washed triple with 100% ethanol, and the particles were kept in a sealed container with 2-propanol. The synthesized nanoparticles were monodisperse (see Fig. 1a,b) with the average size of 100 nm × 150 nm and formed stable colloids in non-polar organic solvents such as hexane, chloroform, etc.

### Emission Spectra

Photoluminescence spectra of UCNPs were recorded by a spectrofluorometer Fluorolog 3 (Horiba JobinYvon, France) with a 975 nm semiconductor laser excitation. PL conversion efficiency (*η*_uc_) of the UCNPs in powder was measured by a calibrated integrating sphere (Labsphere, USA). *η*_uc_ was calculated according to the definition: *η*_uc_ = *P*_em_/*P*_abs_ [W W^−1^]*100%, where *P*_em_ is the emitted power and *P*_abs_ is the absorbed power.

### Transmission Electron Microscopy (TEM) Imaging

High angle annular dark field (HAADF) scanning transmission electron microscopy study was performed using a JEOL ARM200F cold FEG double aberration-corrected electron microscope operated at 200 kV and equipped with a large solid-angle CENTURIO EDX detector and Quantum EELS spectrometer. For TEM examination the nanoparticles were dispersed in methanol with subsequent deposition on a hollow carbon Cu grid.

### Scanning electron microscopy (SEM) Imaging

Scanning electron microscopy imaging of 3D structures and UCNPs was performed using Quanta 400 F scanning electron microscope (FEI Company, Oregon, USA). Before SEM imaging, the laser produced 3D structures were sputter coated with a 10 nm gold layer. For imaging of UCNPs only, the nanoparticles were dispersed in methanol and drop-casted on a glass coverslip. After solvent evaporation, UCNPs on the glass coverslips were sputter coated with 5 nm thin gold layer.

### Photocurable Compositions

Two types of photocurable compositions were prepared. The PCC mixture preparation and polymerization process was performed under yellow illumination, in order to avoid uncontrollable photoininiation induced by blue and UV light.

Polymethacrylates (PMA) are transparent thermoplastics, also known as “acrylic glasses possessing a number of unique features. In particular, their optical properties are superior to those of conventional glasses; these materials are affordable and allow easy handling and processing. The block and the monomer solution polymerization are the most commonly used methods for the PMA preparation characterized by a low initiation rate and a rapid gelation with a significant increase in viscosity, which limits the controllability of the reactions and the properties of the resulting polymer. To overcome this problem, the preparation is performed in two stages. Firstly, the liquid prepolymers are synthesized, while the next step of polymerization results in formation of high-molecular compounds. Such a scenario allows doping of the polymer with the required organic additives such as dyes, plasticizers, etc., and the incorporation of inorganic nanoparticles leading to hybrid organic-inorganic PMA materials. It also worth to mention, that it simplifies the processing required to produce the devices of the desired configuration.

A mixture of equal portions of oligocarbonate methacrylate (OCM-2) and poly(methyl methacrylate) (PMMA) with 1% photoinitiator Irgacure 369 (Ciba Specialty Chemicals Inc.) was used as a photocurable composition (PCC). UCNP dispersion in hexane was added to the photopolymerizable composition and sonicated for 10 minutes. After sonication hexane was evaporated by vacuum drying. PMMA was used to increase the mixture’s viscosity and to prevent nanoparticles’ agglomeration. The final concentration of UCNPs was 15 mg ml^−1^. Then, the mixture was placed into a glass vial as it is shown in Fig. 3c.

For 3D microstructure formation, we used light-curing resin E-shell 300 (EnvisionTEC). E-shell 300 contains diphenyl(2,4,6-trimethylbenzoyl)phosphine oxide as a photoinitiator (commercial name Darocure TPO, Ciba Specialty Chemicals Inc.). UCNP dispersion in dichloromethane was added to the E-shell 300 resin and sonicated for 10 minutes. After sonication, the mixture was placed in a vacuum desiccator to evaporate the solvent. The final concentration of upconversion nanoparticles was 22 mg ml^−1^.

### NIR Triggered Photopolymerization

In order to demonstrate 3D polymeric structure formation in PCC volume, a semiconductor CW laser with a wavelength of 975 nm was used. The collimated laser beam was focused by an objective lens with the focal length of 10 cm into the volume of PCC containing embedded UCNPs. The photopolymerization reaction was proceeding in the local volume of the laser beam caustic, where maximum intensity was achieved. The galvano-scanner (Raylase, Germany), providing fast and precise positioning of the mirrors, was applied for the laser beam deflection in the X-Y-plane. The mirror positioning was controlled using weldMARK 2.0 software. The displacement of voxel along the z-coordinate was carried out by the micrometric stage (Thorlabs, USA). Continuous moving of the laser beam along a circle path was adjusted at the speed of 6 mm s^−1^. After completion of the fabrication, the samples were developed in 2-propanol to remove the unpolymerized PCC material.

The experimental setup for microstructure formation is similar to that recently proposed by us for two-photon polymerization approach^36^. The only difference is that the semiconductor CW laser was used in the current study. A mechanical modulator was applied to trigger the laser light exposure. The X-Y laser position within a small field-of-view was finely controlled by a galvano-scanner (HurryScan10, Scanlab, Germany). The laser beam was passed through a 20 × microscope objective (objective information Zeiss Epiplan × 20, NA: 0.4) and focused into the photosensitive resin. The long-range X-Y adjustment and the sample-objective separation distance were controlled with the piezo-electric translation stages (M-686K004) and a DC-motor (M-126.DG1), respectively (Physik Instrumente (PI) GmbH & Co, Karlsruhe, Germany). A custom-written computer code was developed to produce the experimental microstructures with the desired geometries and the user-defined fabrication parameters. After fabrication, the samples were developed in 1-propanol to remove the unpolymerized material.

### Microbead Formation

The microbead formation procedure was developed for demonstration of the polymerization process at the concentration below the percolation threshold. Firstly, the composition based on E-shell with UCNPs at 0.15 mg ml^−1^ concentration was prepared as described above. Next, this composition was placed in a 1 cm cuvette, and the entire volume was irradiated by scanning laser beam. In order to remove the residual non-polymerized material, the composition was stirred with 1-propanol and centrifuged at 5000 rpm for 10 min. After removing the supernatant, the developing procedure was applied again. As a result, the microbead solution was obtained. After solvent evaporation, the shape of microbeads was studied by SEM microscopy.

## Electronic supplementary material

High-resolution 3D photopolymerization assisted by upconversion nanoparticles for rapid prototyping applications
