# Supplementary material for: High-resolution 3D photopolymerization assisted by upconversion nanoparticles for rapid prototyping applications
Source: Sci Rep. 2018 Feb 26;8:3663. doi: 10.1038/s41598-018-21793-0 (PMC5832145; doi:10.1038/s41598-018-21793-0)
Supplement: Supplementary file 1 — High-resolution 3D photopolymerization assisted by upconversion nanoparticles for rapid prototyping applications [file 41598_2018_21793_MOESM1_ESM.pdf]

Supplementary Information

High-resolution 3D photopolymerization assisted by upconversion nanoparticles for rapid prototyping applications

*Vasilina V. Rocheva, Anastasia V. Koroleva, Alexander G. Savelyev, Kirill V. Khaydukov, Alla N. Generalova, Andrey V. Nechaev, Anna E. Guller, Vladimir A. Semchishen, Boris N. Chichkov and Evgeny V. Khaydukov**

*E-mail: khaydukov@mail.ru

S1. UCNP Emission Spectra

The spectrum of UCNPs corrected for the spectral sensitivity of the Fluorolog 3 HJY is shown in **Fig. S1**.

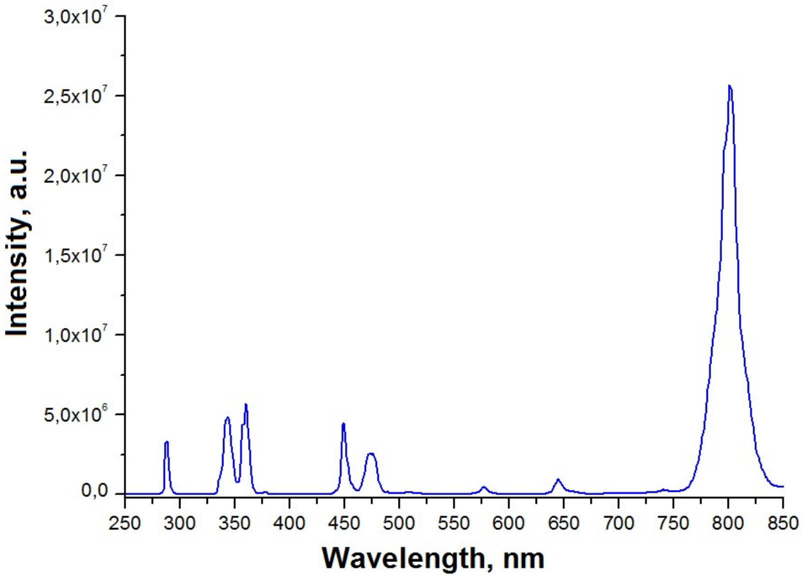

Figure S1. Emission spectrum of the UCNPs *β*-$NaYF_4$:Yb^{3+}, Tm^{3+}/$NaYF_4$ under NIR excitation at 975 nm at intensity 50 W cm^{-2}.

S2. Light Localization in Medium Containing UCNPs

Let us consider the convergent beam propagation in the nonlinear medium uniformly impregnated with upconversion nanoparticles. Assuming the light wave at 975 nm wavelength is not absorbed and not scattered in the material (matrix) of the medium, the probability of nonlinear light absorption by upconversion nanoparticles is defined as:

$$P = \sigma_{\mathrm{N}} I^{\mathrm{N}}, \qquad \text{(S1)}$$

where σ_{N} – nonlinear absorption cross-section, I is intensity at 975 nm wavelength and N is the order of nonlinear absorption (see Fig. S1).

Assuming that the light beam at wavelength λ = 975 nm is Gaussian, the spatial intensity of the beam in cylindrical coordinates can be written as:

$$I(r,z) = I_0 \frac{\omega_0^2}{\omega_{\mathrm{z}}^2} exp\left\{-2\left(\frac{r}{\omega_{\mathrm{z}}}\right)^2\right\}, \qquad \text{(S2)}$$

where ω_0 is the light beam waist, determined at level $1/e^2$, in the lateral plane (x,y).

The beam size in the plane (x,y) can be calculated in any z coordinate as:

$$\omega_{\mathrm{z}} = \omega_0 \sqrt{\left({z\lambda}/{\pi n \omega_0^2}\right)^2 + 1}\,, \qquad \text{(S3)}$$

where n – refractive index of the matrix (medium).

Aiming to define the light beam waist at level $I_0/2$ in z direction, we introduce z_{r}–parameter called also as Rayleigh length:

$$z_{\mathrm{r}} = \frac{\pi n \omega_0}{\lambda}. \qquad \text{(S4)}$$

Taking into account the threshold nature of the photopolymerization process depending on intensity (i.e. concentration of produced radicals equals to the concentration of inhibitors at threshold intensity I_{th}) the effective polymerization voxel size can be found. If we put $I(r,z) = I_{\mathrm{th}}$, we obtain the diameter *D* and the length *L* of the focal region where the intensity exceeds the threshold value[1] and polymerization takes place, according to:

$$D(r) = \omega_0\sqrt{2ln\left(\frac{I(r)}{I_{\mathrm{th}}}\right)}, \qquad \text{(S5)}$$

$$L(z) = 2z_{\mathrm{r}}\sqrt{\frac{I(r)}{I_{\mathrm{th}}} - 1}, \qquad \text{(S6)}$$

Taking into account the nonlinearity of upconversion in the medium with such nanomaterials, the voxel size can be found (by analogy with method of multi-photon polymerization) as:

$$D(r) = \omega_0\sqrt{2ln\left(\frac{I(r)}{I_{\mathrm{th}}}\right)\frac{1}{N}}, \qquad \text{(S7)}$$

$$L(z) = 2z_{\mathrm{r}}\sqrt{\left(\frac{I(r)}{I_{\mathrm{th}}}\right)^{1/N} - 1}, \qquad \text{(S8)}$$

where *N* is the nonlinearity index of the corresponding line, acting in the polymerization process.

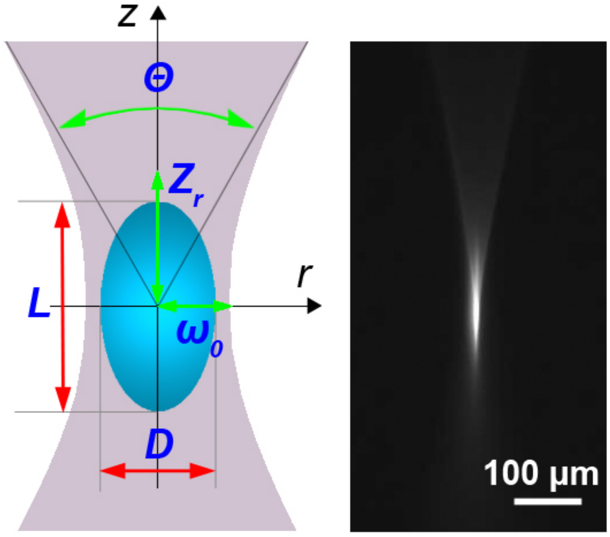

Figure S2. Luminescent voxel formation. A photo of voxel in cuvette containing light-sensitive resin impregnated with UCNPs under the CW NIR light with moderate intensity.

It should also be mentioned that the effective voxel size in the case of NIR-induced polymerization will be slightly higher comparing to that in the two-photon polymerization method due to so called supralinear dependence of NIR light conversion into corresponding PL line[2]. At high intensities of the excitation radiation $P = \sigma_N I^N$ will be converted to $P = \sigma_N I$, leading to voxel size growth.

References

1. Juodkazis, S., Mizeikis, V., Seet, K. K., Miwa, M. & Misawa, H. Two-photon lithography of nanorods in SU-8 photoresist. *Nanotechnology.* **16**, 846 (2005).

2. Khaydukov, E. V., Semchishen, V. A. & Zvyagin, A. V. Incoherent wavefront reconstruction by a retroemission device. *Opt. Lett.* **40**, 1169 (2015).
